# Relative sit-to-stand power cut-off points and their association with negatives outcomes in older adults

**DOI:** 10.1038/s41598-021-98871-3

**Published:** 2021-09-30

**Authors:** Ivan Baltasar-Fernandez, Julian Alcazar, Asier Mañas, Luis M. Alegre, Ana Alfaro-Acha, Leocadio Rodriguez-Mañas, Ignacio Ara, Francisco J. García-García, Jose Losa-Reyna

**Affiliations:** 1 GENUD Toledo Research Group, Universidad de Castilla-La Mancha, Toledo, Spain; 2 CIBER of Frailty and Healthy Aging (CIBERFES), Madrid, Spain; 3 Department of Geriatrics, Hospital Virgen del Valle, Complejo Hospitalario de Toledo, Crta. Cobisa S/N, 45071 Toledo, Spain; 4 Geriatric Department, Hospital Universitario de Getafe, Getafe, Spain

**Keywords:** Ageing, Preventive medicine, Geriatrics, Skeletal muscle

## Abstract

The purposes of this study were: (i) to evaluate the association of sit-to-stand (STS) power and body composition parameters [body mass index (BMI) and legs skeletal muscle index (SMI)] with age; (ii) to provide cut-off points for low relative STS power (STS_rel_), (iii) to provide normative data for well-functioning older adults and (iv) to assess the association of low STS_rel_ with negative outcomes. Cross-sectional design (1369 older adults). STS power parameters assessed by validated equations, BMI and Legs SMI assessed by dual-energy X-ray absorptiometry were recorded. Sex- and age-adjusted segmented and logistic regression analyses and receiver operator characteristic curves were used. Among men, STS_rel_ showed a negative association with age up to the age of 85 years (− 1.2 to − 1.4%^year−1^; *p* < 0.05). In women, a negative association with age was observed throughout the old adult life (− 1.2 to − 2.0%^year−1^; *p* < 0.001). Cut-off values for low STS_rel_ were 2.5 W kg^−1^ in men and 1.9 W kg^−1^ in women. Low STS_rel_ was associated with frailty (OR [95% CI] = 5.6 [3.1, 10.1]) and low habitual gait speed (HGS) (OR [95% CI] = 2.7 [1.8, 3.9]) in men while low STS_rel_ was associated with frailty (OR [95% CI] = 6.9 [4.5, 10.5]) low HGS (OR [95% CI] = 2.9 [2.0, 4.1]), disability in activities of daily living (OR [95% CI] = 2.1 [1.4, 3.2]), and low quality of life (OR [95%CI] = 1.7 [1.2, 2.4]) in women. STS_rel_ declined with increasing age in both men and women. Due to the adverse outcomes related to STS_rel_, the reported cut-off points can be used as a clinical tool to identify low STS_rel_ among older adults.

## Introduction

The proportion of older people is increasing rapidly around the world, and it is expected to reach 16.7% of the total world population by 2050^1^. Importantly, aging is associated with the decline of the neuromuscular system and deterioration of motor function and performance^2,3^. Mechanical power (i.e. product of force and velocity) declines more markedly than other muscular attributes such as muscle mass and strength^4–6^, and has been demonstrated to be an essential concept in geriatric medicine due to its strong relation with negative outcomes^7,8^, but the specific time course of these changes remains poorly understood. In this sense, a recent study conducted in Denmark^9^ showed that allometric (normalized to height^2^), relative (normalized to body mass) and specific (normalized to leg lean mass) power start to decrease importantly from the age of 50 while body mass index (BMI) increases and legs skeletal muscle index (SMI) was maintained up to the age of 75. Woefully, there are no data in Spanish populations and the assessment of mechanical power in older people usually requires expensive instruments (force platform, linear position transducer or 3D accelerometer) that may need periodic calibrations, technical support and can be difficult to transport, which consequently may prevent researchers, clinicians and other health professionals to evaluate mechanical power in daily practice.

Fortunately, the sit-to-stand (STS) muscle power test has emerged as an easy, portable and inexpensive procedure to assess mechanical power in older adults, and seems to be appropriate for the clinical setting^10^. Notably, since most activities of daily living (ADL) require older adults to support their own body mass, relative power has been found to be better associated with impaired physical function, disability and poor quality of life (QoL) than absolute power^5–7,9–11^. Nevertheless, cut-off points to identify older people with a low relative STS power (STS_rel_) have not previously been reported in the literature. In addition, the relationship between low relative power and frailty has been poorly investigated. Frailty is defined as a biological syndrome associated with multisystem declines in physiological reserve and increased vulnerability to adverse outcomes^12^. Since frailty is considered to precede disability (need for assistance to complete ADL^13^), the use of novel instruments strongly associated with frailty that improve the phenotyping of frail older people can be regarded of great relevance. Therefore, the main purposes of the present study were: (i) To evaluate the association of STS power and body composition parameters with age; (ii) to establish cut-off points for low STS_rel_ and its main components according to their ability to discriminate between frail and non-frail older adults; (iii) to assess the association of low STS_rel_ with frailty status, low habitual gait speed, poor QoL and disability in ADL and (iv) to provide normative data for well-functioning older adults.

## Results

The main characteristics of the study participants are shown in Table 1. The percentage of older people with low habitual gait speed (HGS) was 38.8% among men and 57.7% among women; the prevalence of frailty according to the frailty trait scale (FTS) was 13.4% in men and 23.0% in women; and the percentage of people with at least 1 limitation in ADL was 53.4% in men and 30.3% in women.

**Table 1:** Main characteristics of the study participants.

	Men (n = 626)	Women (n = 743)	All (N = 1369)
Age (years)	75.3 ± 6.0	75.6 ± 6.0	75.5 ± 6.0
Body mass (kg)	76.7 ± 11.6	68.8 ± 12.0	72.4 ± 12.5
Height (cm)	163.9 ± 6.5	150.6 ± 5.9	156.7 ± 9.1
BMI (kg m^−2^)	28.5 ± 3.8	30.3 ± 5.1	29.5 ± 4.6
FTS	33.4 ± 13.5	38.9 ± 13.9	36.4 ± 14.0
Katz Index	5.9 ± 0.3	5.8 ± 0.4	5.9 ± 0.4
Lawton Scale	6.7 ± 1.6	7.6 ± 1.1	7.1 ± 1.4
HGS (m s^−1^)	0.89 ± 0.28	0.78 ± 0.25	0.83 ± 0.27
EQ-VAS	78.4 ± 17.9	70.0 ± 21.1	73.8 ± 20.2
EQ-index	0.96 ± 0.07	0.91 ± 0.11	0.93 ± 0.10
Number of medications	4.6 ± 2.7	5.2 ± 2.7	4.9 ± 2.7
Number of diseases	2.6 ± 1.2	3.0 ± 1.2	2.8 ± 1.2
MMSE score	25.0 ± 3.9	24.1 ± 4.1	24.5 ± 4.0

Data are presented as mean ± standard deviation.

*BMI* body mass index, *FTS* frailty trait scale, *HGS* habitual gait speed, *EQ-VAS* EuroQol visual analogue scale, *EQ-index* EuroQol index, *MMSE* mini-mental state examination.

### Association between age and STS power variables

**Figure 1:**
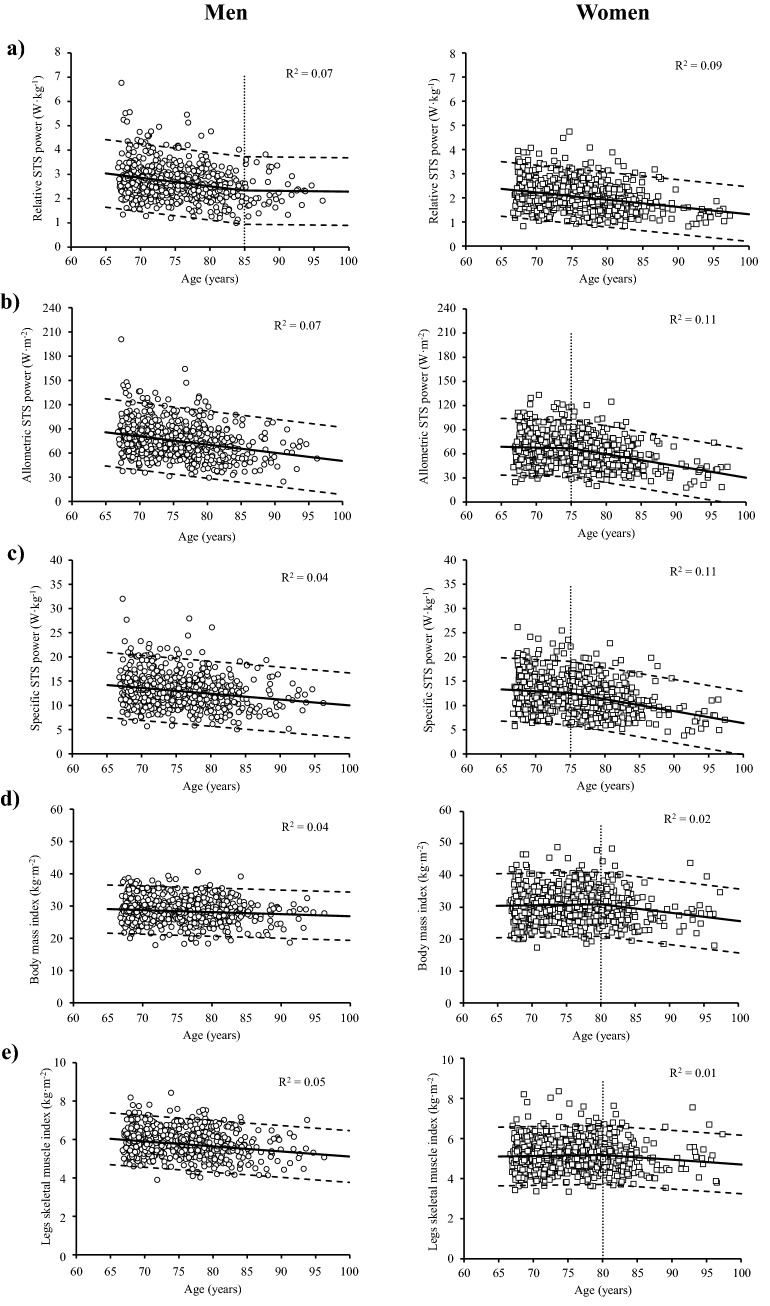
Association between age and relative (**a**), allometric (**b**) and specific STS power (**c**), body mass index (**d**) and legs skeletal muscle index (**e**). Regression lines (continuous lines), 95% confidence intervals (dashed lines), age at which a significant change in slope occurred (dotted lines) and coefficient of determination (R^2^) values obtained from piecewise regression analysis.

Among older men, STS_rel_ showed a negative association with age up to the age of 85 years (between − 1.2 and − 1.4% per year; *p* < 0.05), after which the association was not statistically significant (− 0.1% per year; *p* = 0.257) (Fig. 1a). In older women, STS_rel_ showed a negative association with age throughout the old adult life (between − 1.2 and − 2.0% per year; *p* < 0.001) (Fig. 1a). Allometric and specific STS power showed a negative association in men (between − 1.2 and − 1.8% per year, and between − 0.8 and − 1.1% per year, respectively; both *p* < 0.001) (Fig. 1b,c), while women showed a non-significant negative association between 65 and 75 years old in both parameters (− 0.3% and − 0.6% per year, respectively; both *p* > 0.05) that became statistically significant thereafter (between − 2.2 and − 4.0% per year, and between ‒2.0 and ‒3.3% per year, respectively; both *p* < 0.05) (Fig. 1b,c). There were no significant between-sex differences in terms of annual percentage losses in relative and specific power, while the annual percentage loss in allometric STS power was higher in men compared to women between 65 and 75 years old (*p* < 0.05) (Table 2).

**Table 2:** Annual rate of change (%year^−1^) in muscle power and body composition parameters.

Age group (years)		Relative STS power (W kg^−1^)	Allometric STS power (W m^−2^)	Specific STS power (W kg^−1^)	BMI (kg m^−2^)	Legs SMI (kg m^−2^)
**Men**	n					
65–69	123	− 1.2 ± 0.2*	− 1.2 ± 0.2*^,#^	− 0.8 ± 0.2*	− 0.2 ± 0.1*^,#^	− 0.4 ± 0.1*^,#^
70–74	188	− 1.2 ± 0.2*	− 1.3 ± 0.2*^,#^	− 0.9 ± 0.2*	− 0.2 ± 0.1*^,#^	− 0.4 ± 0.1*^,#^
75–79	166	− 1.3 ± 0.2*	− 1.3 ± 0.2*	− 0.9 ± 0.2*	− 0.2 ± 0.1*^,#^	− 0.5 ± 0.1*^,#^
80–84	105	− 1.4 ± 0.2*	− 1.4 ± 0.2*	− 1.0 ± 0.2*	− 0.2 ± 0.1*^,#^	− 0.5 ± 0.1*
85–89	29	− 0.1 ± 1.2	− 1.5 ± 0.2*	− 1.0 ± 0.2*	− 0.2 ± 0.1*^,#^	− 0.5 ± 0.1*
90–94	13	− 0.1 ± 1.2	− 1.7 ± 0.2*	− 1.1 ± 0.2*	− 0.2 ± 0.1*^,#^	− 0.5 ± 0.1*
95–99	2	− 0.1 ± 1.2	− 1.8 ± 0.3*	− 1.1 ± 0.2*	− 0.2 ± 0.1*^,#^	− 0.5 ± 0.1*
**Women**	n					
65–69	130	− 1.2 ± 0.1*	− 0.3 ± 0.4^#^	− 0.6 ± 0.4	0.1 ± 0.2^#^	0.1 ± 0.1^#^
70–74	212	− 1.3 ± 0.1*	− 0.3 ± 0.4^#^	− 0.6 ± 0.4	0.1 ± 0.2^#^	0.1 ± 0.1^#^
75–79	220	− 1.4 ± 0.2*	− 2.2 ± 0.6*	− 2.0 ± 1.1*	0.1 ± 0.2^#^	0.1 ± 0.1^#^
80–84	132	− 1.5 ± 0.2*	− 2.5 ± 0.7*	− 2.2 ± 1.2*	− 0.9 ± 0.6*^,#^	− 0.5 ± 0.3
85–89	29	− 1.7 ± 0.2*	− 2.9 ± 0.8*	− 2.5 ± 1.4*	− 0.9 ± 0.7*^,#^	− 0.5 ± 0.3
90–94	12	− 1.8 ± 0.2*	− 3.3 ± 1.0*	− 2.8 ± 1.6*	− 0.9 ± 0.7*^,#^	− 0.5 ± 0.3
95–99	8	− 2.0 ± 0.2*	− 4.0 ± 1.2*	− 3.3 ± 1.9*	− 1.0 ± 0.7*^,#^	− 0.5 ± 0.3

*STS* sit-to-stand, *BMI* body mass index, *legs SMI* legs skeletal muscle index.

*Significantly different compared with a slope equal to zero (*p* < 0.05).

^#^Significant differences between women or men at the same age (*p* < 0.05).

### Association between age and body composition variables

Both BMI and legs SMI values showed a negative association with age throughout the old adult life among men (− 0.2% per year, and between − 0.4 and − 0.5% per year, respectively; both *p* < 0.05) (Fig. 1d,e). In contrast, older women reported a non-significant positive association with age in both BMI and legs SMI between 65 and 80 years old (both 0.1% per year; both *p* > 0.05), followed by a significant negative association in BMI (between − 0.9 and − 1.0% per year, *p* < 0.05) but not in legs SMI (− 0.5% per year; *p* > 0.05) (Fig. 1d,e). In terms of BMI, higher annual percentage losses in older men compared to older women between 65 and 80 years old were noted, while the opposite occurred from 80 years to oldest age (all *p* < 0.05). Regarding legs SMI, significant between-sex differences were only noted between 65 and 80 years old, with older men showing a greater annual percentage loss than older women (*p* < 0.05) (Table 2).

### Cut-off values

**Figure 2:**
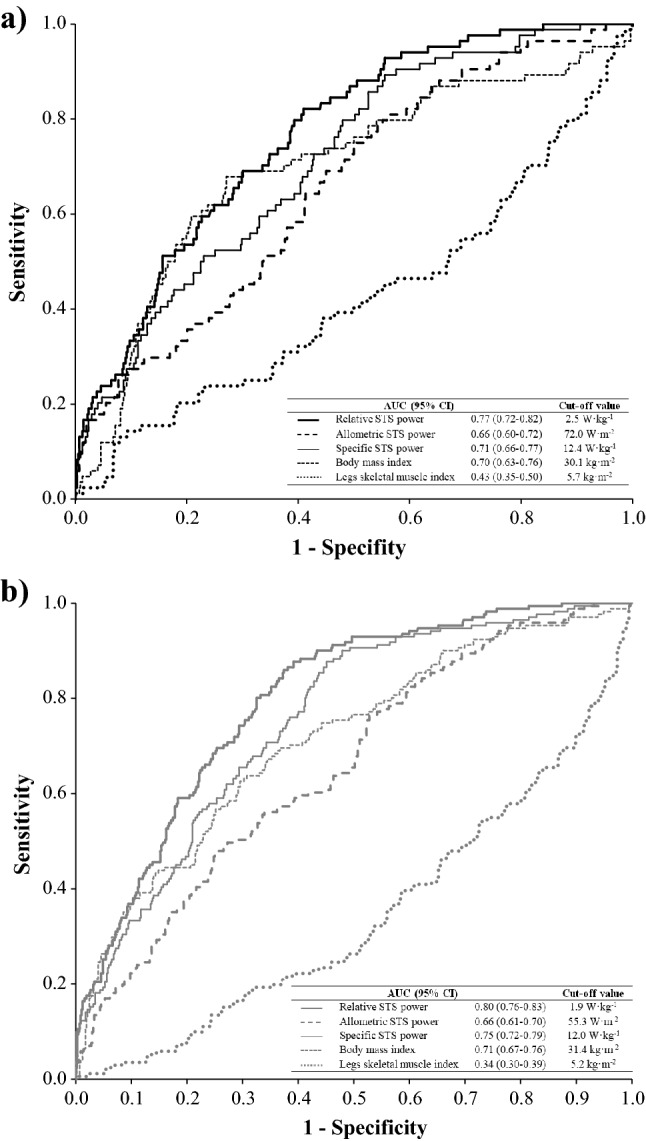
Receiver operator characteristic curves showing the ability of STS power and body composition measurements to discriminate between frailty status in men (**a**) and women (**b**) with their specific cut-off values.

Receiver operator characteristic (ROC) curves, area under the curve (AUC) values and calculated relative, allometric and specific STS power, BMI and SMI cut-off values for discriminating frailty from those without frailty are displayed for older men and women in Fig. 2.

### Association between low STS_rel_ power and negative outcomes

**Figure 3:**
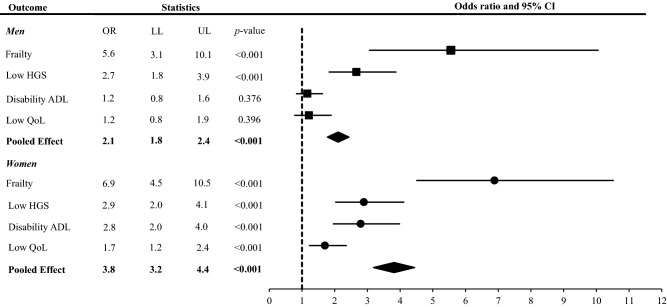
Association between low relative STS muscle power and negative outcomes. The analysis was adjusted by age. Pooled effect size was calculated with frailty (FTS), low HGS, disability ADL and Low Qol. *Low HGS* low habitual gait speed, *Disability ADL* disability in one or more activities of the daily living (instrumental and basic combined), *Low Qol* low quality of life.

Older adults with low STS_rel_ (≤ 2.5 and ≤ 1.9 W kg^−1^ for men and women, respectively) showed higher odds of being frail (OR [95% CI] = 5.6 [3.1–10.1]) and having low HGS (< 0.8 m s^−1^) (OR [95% CI] = 2.7 [1.8–3.9]) when compared to older men with normal STS_rel_. No association of low STS_rel_ with disability in ADL was noted among older men (*p* > 0.05). For older women, low STS_rel_ was associated with frailty (OR [95% CI] = 6.9 [4.5–10.5]), low HGS (OR [95% CI] = 2.9 [2.0–4.1]), disability in ADL (OR [95% CI] = 2.8 [2.0–4.0]), and low QoL (OR [95% CI] = 1.7 [1.2–2.4]). The pooled effect size of low STS_rel_ on the recorded negative outcomes was statistically significant in both older men (OR [95% CI] = 2.1 [1.8–2.4]) and older women (OR [95% CI] = 3.8 [3.2–4.4]) (Fig. 3).

### Normative values for well-functioning older participants

**Figure 4:**
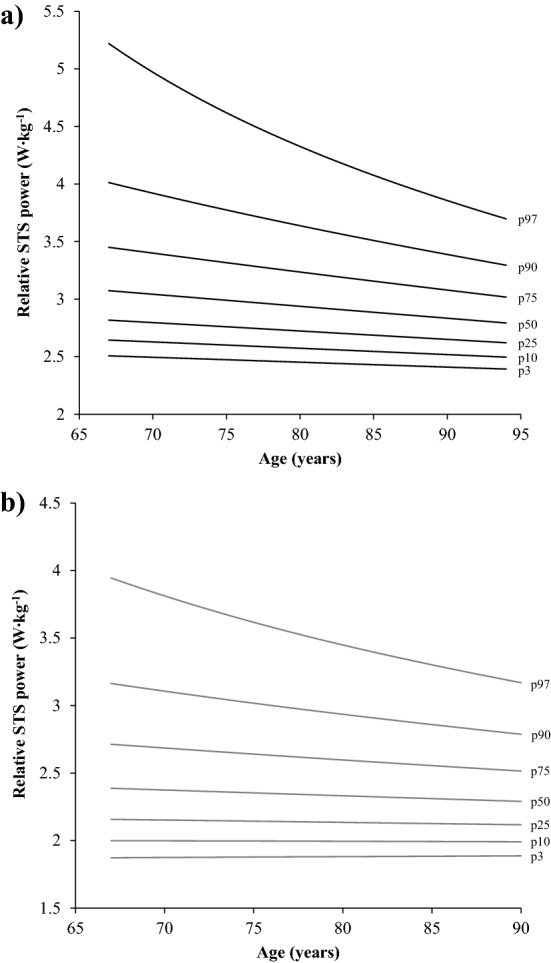
Normative values of relative STS power in men (**a**) (n = 337) and women (**b**) (n = 413) with relative STS power values above the identified thresholds of ≥ 2.5 and 1.9 W kg^−1^ for men and women, respectively. *STS* sit-to-stand, *p* percentile.

A total of 338 (54.0%) men and 417 (56.1%) women had STS_rel_ above the reported cut-off values (≥ 2.5 and 1.9 W kg^−1^ for men and women, respectively). Smoothed age-specific normative values for well-functioning older men and older women are shown in Fig. 4. In addition, sex-specific STS_rel_ values corresponding to percentiles 3, 10, 25, 50, 75, 90 and 97 are shown in Table 3.

**Table 3:** Smoothed age-specific percentiles of relative STS power (W kg^−1^) in men and women with relative STS power values above the identified thresholds of ≥ 2.5 and 1.9 W kg^−1^ for men and women, respectively.

Sex	n	P3	P10	P25	P50	P75	P90	P97
Men	337	2.5	2.6	2.7	2.9	3.2	3.6	4.3
Women	413	1.9	2.0	2.1	2.3	2.6	3.0	3.5

*P* Percentile.

## Discussion

The main findings of the present investigation using a large sample of Spanish older adults (n = 1369) were: (i) in older men STS_rel_ significantly declined until the age of 85 years as result of the loss of specific STS power and legs SMI; (ii) in older women STS_rel_ significantly declined until the oldest age due to the loss of specific STS power and the increase in BMI before the age of 80 years, and due to the loss of specific STS power and legs SMI after the age of 80 years; and (iii) low STS_rel_ (< 2.5 W kg^−1^ in men and < 1.9 W kg^−1^ in women) was associated with frailty and impaired physical function in older men, and with frailty, impaired physical function, disability in ADL and poor QoL in older women.

### Age-related changes in power and body composition parameters

STS_rel_ has been shown to decline after the age of 40 years in both women and men^9^. This age-related decline in relative power was caused by the loss of specific power and the increase in BMI before the age of 65 years in men and 75 years in women, and by the loss in both specific power and legs SMI thereafter^9^. Those findings are compatible with the findings shown in the present study regarding the earlier decline in BMI and legs SMI in older men compared to older women (above 65 vs. 80 years, respectively). Consequently, our reported mechanisms accounting for the loss of STS_rel_ above the age of 65 years and their sex-specific timing coincide with those previously found by Alcazar et al.^9^ in a Danish older population. However, we observed no further decline in STS_rel_ among men older than 85 years. This may be due to older men below a certain level of relative power being less likely to be alive^14^ or to be willing to participate in the current investigation (i.e. survival effect). On the other hand, the percentage changes in STS_rel_ noted in the present study (women: − 1.2 to − 2.0% per year; men: − 0.1 to − 1.4% per year) were similar to those evidenced by other investigations: − 1.7% per year^15^ and − 1.5–3.1% per year^9^.

### Cut-off points for low STS_rel_ and their association with negative outcomes

The relevance of relative power has been evaluated by several studies demonstrating that lower levels of relative power are associated with low physical function^7,10,16–18^, poor cognitive function and health-related QoL^10,18^, higher risk of falls^19^, dependence in ADL^20,21^ and mortality^14^. However, the association between relative power and frailty in older adults remains unknown. To our knowledge, only one previous study has evaluated the relationship between mechanical power and frailty^18^. Specifically, that study showed that having lower levels of STS_rel_ was associated with several negative outcomes (frailty, decreased gait speed, disability and poor quality of life) than sarcopenia^18^. However, in the above-mentioned study low STS_rel_ was based on sex-specific tertiles instead of using a more objective approach to determine the cut-off points for low relative power. For that reason, in the current investigation we found those cut-off points for low STS_rel_ that better discriminated between people with and without frailty, due to the latter is reported to accompany by poor gait ability, fatigue, higher risk of falls and overall difficulty to perform activities of daily living^22^. Specifically, cut-off points for low STS_rel_ were 2.5 W kg^−1^ in men and 1.9 W kg^−1^ in women. Thus, older people below these cut-off points were more likely to be frail and to have a low HGS, while additionally; older women below the cut-off point were also more likely to present disability in ADL and poor QoL. The lack of association between low STS_rel_ and disability in ADL in older men is in accordance with the results found by Kozicka and Kostka^20^ who also noted a significant association between relative power and the performance in ADL in women but not in men. This phenomenon could be explained by the existing inequality between men and women in the participation in household chores, which is on average, more than double in women compared with men^23^. Thus, some older men with adequate levels of muscle power may present lower ADL scores because they do not participate in ADL voluntarily as a consequence of traditional gender roles. This aspect would affect (decrease) the association values found between low muscle power and ADL disability in men. Another explanation might be the extended longevity of women compared with men at all ages, so as long as men are fully independent, they live longer than women, but once their health begins to deteriorate, the progression of disability and the onset of death are faster in men^24^.

### Normative values of STSrel in older people above the reported cut-off points

Apart from the necessity of discriminating between older people with and without low STS_rel_ due to the negative consequences derived from the former, the provision of normative data to older people with adequate levels of STS_rel_ may also be of great relevance from a prevention perspective. This strategy may help (a) older people to be aware of their levels of relative power when compared with their counterparts and (b) health professionals to detect older people with decreased (albeit normal) levels of STS_rel_ who are in time to postpone the onset of low STS_rel_ and its derived negative consequences. Only one previous study^21^ has reported normative values for relative peak power exerted during countermovement jumps performed over a force plate in older adults. Nevertheless, performing countermovement jumps may not be feasible in a relatively large proportion of older people (especially those > 80 years old) and the use of a force plate may not be available in the clinical setting. Fortunately, the STS muscle power test and the cut-off points and normative data provided in the current study constitute a feasible and clinically relevant strategy to be applied in older people in the clinical setting or other health-related settings.

### Study limitations

The cross-sectional design of the present study may have influenced our results by means of a survival effect bias; however, identical limitations are shown in longitudinal designs^25^. Moreover, although computed tomography and magnetic resonance imaging has been demonstrated to be gold standards to assess human skeletal muscle mass in vivo, we evaluated legs appendicular lean mass with dual energy X-ray absorptiometry (DXA), which has been recognized as a recommended option in the clinical setting^26^. Finally, we should point out that this study included a large sample of Spanish older adults with objective measurements of STS power and body composition, which provide relevant information about the different trajectories of these measurements throughout old age. Of note, the main practical application is that the STS muscle power test seems a feasible and valid test to assess relative muscle power, which can be classified as low and normal according to the cut-off points provided in the present manuscript. Then, the different underlying components of relative muscle power can be determined in order to find out the reason(s) why an older adult presents low relative muscle power.

## Conclusions

STS_rel_ was observed to decline until the age of 85 years as a result of the loss of specific STS power and legs SMI in men. In women, STS_rel_ declined until the oldest age due to the loss of specific STS power and the increase in BMI before the age of 80 years, and due to the loss of specific STS power and legs SMI after the age of 80 years. STS_rel_ values below 2.5 W kg^−1^ in men and 1.9 W kg^−1^ in women were strongly related with low HGS and frailty in both men and women and with low QoL and disabilities in ADL only in women. Moreover, this study provided normative values for well-functioning older adults. This information can be of high relevance to health-related professionals interested in the detection of older adults with low STS_rel_, who may benefit the most from power-based resistance training interventions to preserve power and reduce the risk of frailty.

## Materials and methods

### Design

This investigation considered data from the Toledo Study for Healthy Ageing (TSHA), a Spanish population-based prospective cohort study involving men and women over 65 years whose main goal was to examine the prevalence and underlying mechanisms of the frailty syndrome. Specifically, the present study includes cross-sectional data collected from the participants from 2011 to 2013. Full methodology of the TSHA has been previously described elsewhere^27,28^.

The study protocol was approved by the Clinical Research Ethics Committee of the Toledo Hospital, Spain. This work was performed according to the ethical standards laid down in the 1964 Declaration of Helsinki and later amendments.

### Participants

Study participants were selected by a two-stage random sampling from the municipal census of Toledo from rural and urban settings. After excluding older participants who did not perform the physical performance or body composition measurements, a total of 1369 older adults (743 women and 626 men; 66.7–97.3 years) were included in the analysis (Table 1). Information on number of medications, number of diseases and cognitive function as assessed with Mini-Mental State Examination (MMSE)^29^ was also included for a better understanding of the study participants’ characteristics. Participants signed informed consent forms prior to their inclusion in the study.

### Anthropometrics and body composition parameters

Standard anthropometric assessment (height and body mass) was conducted by a stadiometer and scale device (Seca 711, Hamburg, Germany). BMI was calculated as body mass divided by height^2^ (kg m^−2^). Body composition was assessed by DXA (Hologic, Serie Discovery QDR, Bedford, USA) and analysed with commercially available software (Physician’s Viewer, APEX System Software Version 3.1.2., Bedford, USA). The participants were studied in the supine position, wearing light clothing with no metal, shoes or jewellery. A regional analysis was conducted in order to obtain legs lean mass. Legs SMI was calculated as legs appendicular lean mass divided by height^2^ (kg m^−2^).

### Mechanical power evaluation

Mechanical power was assessed during the 5-rep STS test using the equations validated by Alcazar et al.^10^. After the cue “ready, set, go!” the participants started to perform 5 timed STS repetitions as rapidly as possible on a 0.43 m chair from the sitting position with their buttocks touching the chair to the full standing position, with their arms crossed over the chest. They performed a first practice attempt followed by a minimum of two valid trials with 2 min of rest between trials. Time to complete the task (from the cue “go!” until the participant sat down after the fifth STS repetition) was recorded using a stopwatch to the nearest 0.01 s. The fastest attempt was chosen for the analysis. Then, the following equation was used as previously reported^10^:

$$\begin{aligned} & STS\,power \\ & \quad = \frac{{Body\,mass \left( {{\text{kg}}} \right) \times 0.9 \times {\text{g}} \times \left[ {Height \left( {\text{m}} \right) \times 0.5 - Chair height \left( {\text{m}} \right)} \right]}}{{STS time \left( {\text{s}} \right) \times STS\, repetitions^{ - 1} \times 0.5}}. \\ \end{aligned}$$

Of note, variations in relative muscle power have been found to be motivated by changes in its underlying components^9^: allometric power, BMI, specific power and legs SMI. In order to assess the relationship between age and the different components accounting for relative STS power on frailty, relative STS power and their main components were calculated as follows^9^: (1) relative STS power (W kg^−1^) was calculated as the ratio of STS power and body mass; (2) allometric STS power (W m^−2^) was calculated as the product of relative power and BMI; and (3) specific STS power (W kg^−1^) was calculated as the ratio between allometric power and legs SMI. Of note, due to the association observed between absolute muscle power and body size (r = 0.48 in men and r = 0.47 in women)^30^, allometric muscle power (absolute muscle power normalized to height squared) was calculated and used in further analyses.

### Frailty, low physical function, disability in activities of daily living and low quality of life

Frailty was assessed according to the FTS, which evaluates seven different dimensions (energy balance and nutrition, physical activity, nervous system, vascular system, weakness, endurance and slowness) and provides a score between 0 and 100 points. A FTS score ≥ 50 represented frailty^31^.

Physical function was evaluated by the 3-m habitual gait speed test. Participants were asked to walk at their habitual gait speed along a 3-m distance. The time needed to complete the distance was recorded with a stopwatch to the nearest 0.01 s and the best time of two attempts was chosen for further analysis. Low HGS was defined as a HGS lower than 0.8 m s^−1^^32^.

Disability in instrumental activities of daily living (IADL) and basic activities of daily living (BADL) were evaluated by Katz index^33^ and Lawton and Brody Scale^34^, respectively. Disability in ADL was considered if one or more limitations were reported for any IADL or BADL.

Finally, health-related quality of life was measured using the EQ-5D-5L questionnaire. Briefly, the participants rated their difficulties in five dimensions (mobility, self-care, usual activities, pain/discomfort and anxiety/depression) and the EQ-5D-5L index (EQ-index) was calculated based on the crosswalk value set from the Spanish time trade-off valuation technique by using the EQ-5D-5L Crosswalk Index Value Calculator^35^. Moreover, the participants’ self-reported health-state was assessed by the EuroQol visual analogue scale (EQ-VAS). Low QoL was defined as the lowest sex-specific tertiles of both EQ-index (< 1.00 for men and < 0.91 for women) and EQ-VAS (< 70 for men and < 50 for women).

### Statistical analysis

All analyses were performed separately in men and women. Continuous variables are reported as mean ± standard deviation (SD) and categorical variables as percentage. The relationship between age and STS power (relative, allometric and specific STS power) and body composition (BMI and legs SMI) parameters were assessed by segmented (piecewise) regression analyses^9^. In order to detect potential age points at which there was a change in slope, we used an iterative approach by which different age points (70, 75, 80, 85, 90, and 95) across various age intervals (65–75, 70–80, 75–85, 80–90, 85–95 and 90–100, respectively) were assessed. Only those age points at which a significant change in slope occurred were included in the final segmented regression model. Then, differences in regression slopes between men and women were evaluated by including an age-by-sex interaction term within the regression model. Pairwise comparisons were adjusted by Bonferroni’s correction to reduce multiple testing-derived type I error. ROC curves were analysed to obtain optimal cut-off values for low relative, allometric and specific STS power, high BMI and low legs SMI, according to their ability to discriminate between frail and non-frail older people. Briefly, AUC values were reported, and cut-off values were calculated based on the best trade-off (product) between sensitivity and specificity. Sex-specific and age-adjusted logistic regression models were used to assess the association of low STS_rel_ with various negative outcomes: frailty, low HGS, disability in ADL and low QoL. Besides, the pooled effect size (the combined effect on all above-mentioned negative outcomes) of low STS_rel_ was calculated.

Finally, to provide relevant clinical information also to those older participants with STS_rel_ above the sex-specific cut-off points, normative data were calculated for a sub-sample of older adults with adequate levels of STS_rel_ using the LMS method^36^ and a specialized software (LMS Chart Maker Light 2.5, Medical Research Council, UK). The LMS method provides a way of normalized growth percentile standards. The model is constructed by transforming data of those age groups with skewed distribution into an appropriate normal distribution of non-biasedness using three parameters: L (Box-Cox transformation), M (median) and S (coefficient of variation)^37^. The appropriate number of degrees of freedom was selected according to LMS chartmaker guidelines. Age- and sex-specific percentiles 3, 10, 25, 50, 75, 90 and 97 were reported. Statistical analyses were performed using SPSS v23 (SPSS Inc., Chicago, Illinois) and significance was assessed with a two-tailed α level of 0.05.
